# A Spatio-Temporal Capsule Neural Network with Self-Correlation Routing for EEG Decoding of Semantic Concepts of Imagination and Perception Tasks

**DOI:** 10.3390/s24185988

**Published:** 2024-09-15

**Authors:** Jianxi Huang, Yinghui Chang, Wenyu Li, Jigang Tong, Shengzhi Du

**Affiliations:** 1 School of Electrical Engineering and Automation, Tianjin University of Technology, Tianjin 300384, China; 18562669812@163.com; 2 First Teaching Hospital of Tianjin University of Traditional Chinese Medicine, Tianjin 300193, China; byh_beilei@163.com; 3 National Clinical Research Center for Chinese Medicine Acupuncture and Moxibustion, Tianjin 300381, China; 4 College of Artificial Intelligence, Nankai University, Tianjin 300350, China; 5 Department of Electrical Engineering, Tshwane University of Technology, Pretoria 0001, South Africa

**Keywords:** brain-computer interface (BCI), EEG decoding, semantic concepts, capsule neural network, self-correlation routing

## Abstract

Decoding semantic concepts for imagination and perception tasks (SCIP) is important for rehabilitation medicine as well as cognitive neuroscience. Electroencephalogram (EEG) is commonly used in the relevant fields, because it is a low-cost noninvasive technique with high temporal resolution. However, as EEG signals contain a high noise level resulting in a low signal-to-noise ratio, it makes decoding EEG-based semantic concepts for imagination and perception tasks (SCIP-EEG) challenging. Currently, neural network algorithms such as CNN, RNN, and LSTM have almost reached their limits in EEG signal decoding due to their own short-comings. The emergence of transformer methods has improved the classification performance of neural networks for EEG signals. However, the transformer model has a large parameter set and high complexity, which is not conducive to the application of BCI. EEG signals have high spatial correlation. The relationship between signals from different electrodes is more complex. Capsule neural networks can effectively model the spatial relationship between electrodes through vector representation and a dynamic routing mechanism. Therefore, it achieves more accurate feature extraction and classification. This paper proposes a spatio-temporal capsule network with a self-correlation routing mechaninsm for the classification of semantic conceptual EEG signals. By improving the feature extraction and routing mechanism, the model is able to more effectively capture the highly variable spatio-temporal features from EEG signals and establish connections between capsules, thereby enhancing classification accuracy and model efficiency. The performance of the proposed model was validated using the publicly accessible semantic concept dataset for imagined and perceived tasks from Bath University. Our model achieved average accuracies of 94.9%, 93.3%, and 78.4% in the three sensory modalities (pictorial, orthographic, and audio), respectively. The overall average accuracy across the three sensory modalities is 88.9%. Compared to existing advanced algorithms, the proposed model achieved state-of-the-art performance, significantly improving classification accuracy. Additionally, the proposed model is more stable and efficient, making it a better decoding solution for SCIP-EEG decoding.

## 1. Introduction

Brain-computer interface (BCI) is one of the current research hotspots, which can achieve direct communication of the brain with the external world without relying on muscles or the nervous system. Therefore, BCI enables users to control external devices such as computers, wheelchairs and prostheses through brain activities [1,2,3]. BCI technology has been widely applied in many fields such as medical treatment [4], rehabilitation [5,6], intelligence control [7], augmented reality [8], virtual reality [9] and neuroscience research [10], and has shown new potentials and development prospects. In BCI, various methods are used to present biological signals, with EEG being the most common choice for brain signal decoding due to its high temporal resolution, non-invasiveness, relatively low cost, and portability. EEG-based BCI systems use different paradigms, including event-related potentials (ERPs), steady-state visual evoked potentials (SSVEPs) [11], and motor imagery [12]. Oscillatory activities in tasks, such as sleepiness, can be effectively detected through EEG signals. Recently, decoding of semantic concepts for imagination and perception (SCIP) has attracted increasing interest. Decoding semantic information has the advantage of being able to generalize the main concepts of a task [13]. For example, when presenting an image, instead of focusing on low-level sensory details such as the colour and the shape, people also focus on the high-level semantic concepts of the object in the image. Visual decoding is often interfered with by irrelevant information such as shape, size, colour, etc., whereas semantic decoding extracts conceptual information including object type or category. The capability of ignoring low-level sensory details is considered a desirable quality for BCI systems. There is growing evidence showing that imagination and perception overlap at the neural level [14]. This overlap may facilitate the feasibility of cross-task decoding [15]. This neural overlap between imagination and perception can be explored by analysing how different sensory inputs (e.g., pictorial, orthographic and audio) are processed by the brain during perception and imagination. This research has opened up the possibility of developing more robust BCI systems, which are important for the understanding of the neural mechanisms of semantic concepts during imagination and perception in the field of rehabilitation [16,17]. However, EEG signals are prone to significant noise and interference, making the decoding of SCIP-EEG signals a critical challenge to address.

Initial feature extraction from EEG signals can be categorized into time-domain [18], frequency-domain [19], and spatial-domain [20] and some multi-domain combination methods [21,22,23]. Frequency-domain features examine the energy distribution across different frequencies, using Fast Fourier Transform [24], wavelet transform [25], autoregression, and other spectral analysis methods. Spatial-domain analysis uses spatial mapping techniques to project EEG data to spatial distributions in the manner to enhance feature classification, where Common Spatial Patterns (CSP) [26] are commonly used. The extracted features are then classified using machine learning methods, such as SVM (Support Vector Machine) [27], LDA (Linear Discriminant Analysis) [28], and KNN (K-Nearest Neighbors) [29]. SVM is a binary classification model that maps feature vectors to a two-dimensional space aiming to find an optimal dividing curve that effectively differentiates feature vectors. LDA is a traditional binary classification algorithm that reduces dimensionality of features and projects them onto a one-dimensional coordinate system for classification through optimal thresholds. KNN is suitable for multi-class tasks and determines the class of features via its distance to known class samples and using majority voting within a certain range. Additionally, various other traditional machine learning methods are used for EEG signal classification.

With the advent of deep learning [30], multiple deep neural network architectures have been applied to EEG signal feature extraction and classification. Deep learning, compared to traditional machine learning, shows greater capability in EEG feature extraction. Convolutional Neural Networks (CNNs) were among the earlier deep learning models used for decoding EEG signals in BCIs [31,32,33]. However, due to the limitations of CNNs, current applications have nearly reached their maximum potential. Subsequently, Recurrent Neural Networks (RNNs) [34] and Long Short-Term Memory networks (LSTMs) [35] were introduced in the BCI field, though they are primarily suited for time series prediction, instead of EEG signal decoding. In RNNs and LSTMs, the transformer model [36] effectively addressed the importance of parallel computation and capturing long-distance features. For EEG signals, the available datasets are usually small and may not be sufficient to adequately train the deep learning models, such as Transformer. The high spatial correlation characteristic makes EEG signals suitable for Capsule Neural Networks [37]. Capsule Neural Networks exhibit several advantages in EEG signal classification:

- Firstly, the capsule network utilizes the directional properties of its units. This is helpful to effectively sense and resolve the EEG signals in different postures, because the brainwave characteristics vary depending on the head position or the angle of electrode placement.
- Secondly, the multilayer capsule structure of Capsule Neural Networks allows for a hierarchical representation of features so that each unit can accurately represent features at different levels.
- Thirdly, the dynamic routing mechanism can learn and optimize the signal correlations across different electrodes, time domain features, frequency domain features.

**Figure 1:**
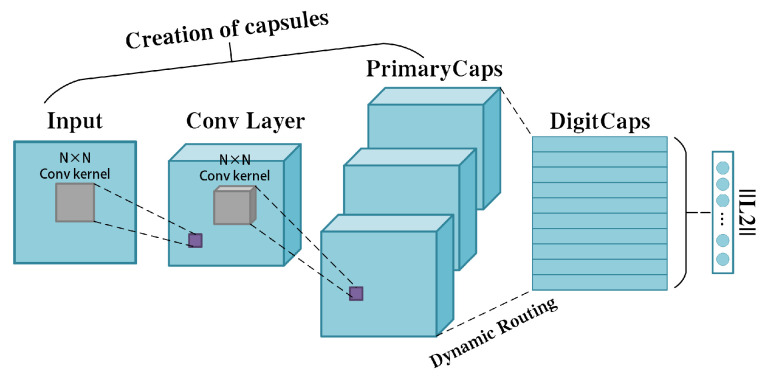
The structure of capsule neural networks.

Typically, capsule neural network is divided into three parts: the convolutional layers, the primary capsule layers, and the digital capsule layers. First, the convolutional layers are used to extract features and instantiate and encode the input vectors. Then, the primary capsule layers receive the feature representations from the convolutional layers and organize them into capsules. The capsule is the core unit of the capsule neural network, consisting of vectors or a matrix formed by a group of neurons. These neurons collectively represent various attributes of a specific object, such as position and orientation. Unlike traditional neural networks where each neuron outputs a single scalar value, a capsule outputs a vector. Finally, the representation generated by the primary capsule layers is fed to the digital capsule through a dynamic routing mechanism to obtain the desired output. Most of the previous capsule neural networks for decoding EEG signals [38,39,40,41,42] create capsules in the manner shown in Figure 1. However, EEG signals have complex dynamic characteristics in both time and spatial domains. Using N-by-N convolutional kernels may not be able to capture important features in both time and spatial domains, leading to the loss of information. Therefore, this method cannot efficiently represent the features of the EEG signals well in capsule form. Combining the properties of EEG signals, we can improved the feature extraction part of the capsule neural network. The spatio-temporal features of the EEG signals are extracted using a convolutional neural network. The extracted spatio-temporal features are stored in capsules, which are different than the traditional instantiated attributes. These capsules are called spatio-temporal capsules. In addition, the traditional dynamic routing mechanism undergoes complex computations to update the weights between capsules, which increases the overall complexity of the model and lacks connections between capsules. In the proposed method in this paper, we replace dynamic routing with a novel non-iterative, highly parallel routing mechanism. Overall, compared to previous capsule-based neural network methods, our proposed method makes improvements on capturing the spatio-temporal features of EEG signals, optimising the inter-capsule routing mechanism, and improving the model efficiency and stability. These advantages make the proposed method more suited to handle complex SCIP-EEG-like decoding tasks. The main contributions of this paper are as follows:

1. Improved the feature extraction part of the capsule neural network. The convolutional neural network is used to extract the spatio-temporal features in the EEG signals, replacing the traditional instantiated attributes with spatio-temporal features, which are more adaptive to the complex dynamic characteristics of EEG signals.
2. A novel non-iterative routing mechanism called the self-correlation routing mechanism is proposed. This routing mechanism allows the model to compute similarities between different capsules and assign different similarity weights based on these similarities. This routing mechanism efficiently captures key features and improves the robustness and performance of the model. Compared to traditional dynamic routing, it is non-iterative and highly parallel, which reduces the model complexity while establishing better connections between capsules.
3. Validation was carried out on a publicly accessible EEG-based BCI dataset for imagination and perception tasks of semantic concepts from the University of Bath. We classified perception and imagination tasks across three different sensory modalities (pictorial, orthographic, and audio), achieving average accuracies of 94.9%, 93.3%, and 78.4%, respectively, with an overall average accuracy of 88.9%. Compared to existing advanced algorithms, the proposed model significantly enhances classification accuracy and demonstrates superior performance over state-of-the-art methods.

The rest of this paper is organized as follows. Section 2 introduces the related work on the differences between capsule neural networks and traditional neural networks. Section 3 provides a detailed description of the proposed method. Section 4 describes the dataset and discusses the experimental results, the sensitivity of the parameters, and the ablation study. In Section 5, we summarize the paper.

## 2. Related Work

The basic building blocks of a neural network are neurons. By connecting a large number of neurons, complex network structures are formed. In traditional neural networks, each neuron receives the output of neurons in the previous layer in the form of a weighted summation. The result is then transformed nonlinearly by an activation function to produce an output of the neuron. With the advent of deep learning techniques, more complex and efficient neural network structures were explored. Capsule neural networks are one such innovative structure. Unlike traditional neurons, the basic units of a capsule network are capsules, representing the output of a group of neurons in vector form. The magnitude of a capsule indicates the probability of an entity’s presence, while its direction describes the entity’s attributes. The connection weights in a capsule network are in matrix form, which enables them to describe the relationships and influences between capsules. Capsule networks are designed to overcome the limitations of traditional neural networks in processing spatial relationships. For example, they are particularly suited to handling changes in object poses and partial occlusions in images, thanks to their vector-form attribute representation and enhanced spatial relationship modeling capabilities.

## 3. Methods

### 3.1. Overall Architecture

**Figure 2:**
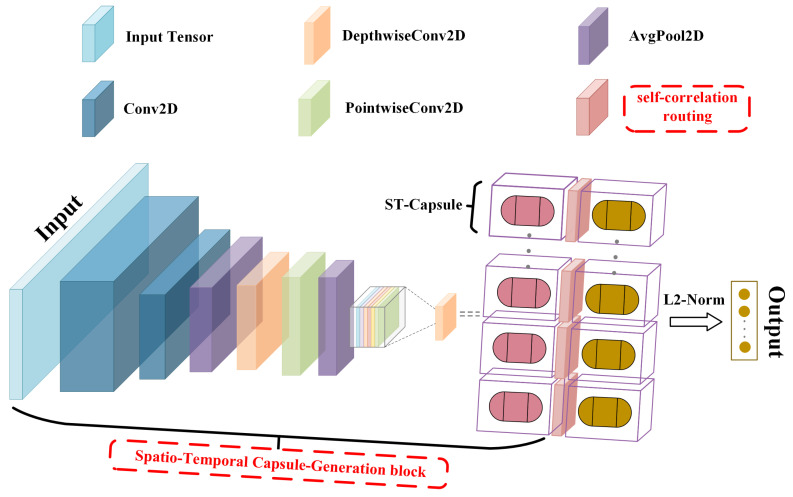
The overall structure of the proposed Efficient-STCapsNet, consisting of two parts: Spatio-Temporal Capsule-Generation block for creating spatio-temporal capsules and self-correlation routing of spatio-temporal capsules.

**Figure 3:**
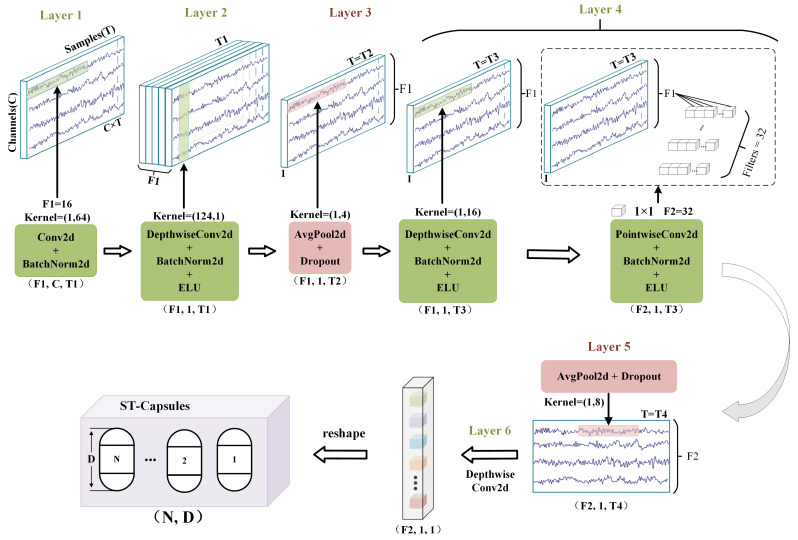
The starting phase consists of five convolutional layers and two pooling layers that will extract temporally and spatially significant features in the EEG signal to generate capsules ST-Capsules, or $s_{n,d}^{l}$ for short (*l* is the number of layers, *n* denotes the number of capsules, and *d* denotes the dimensionality of the capsule).

**Figure 4:**
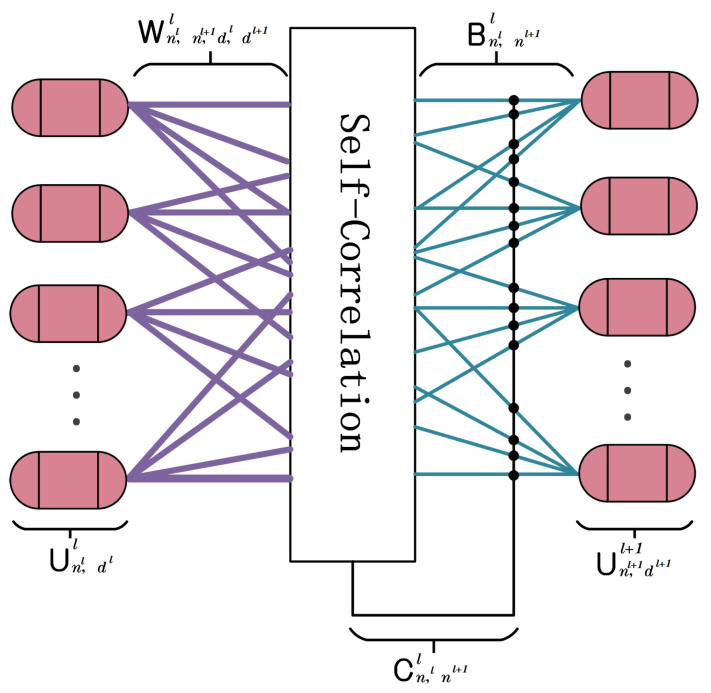
The capsules in layer *l* + 1 further predict the overall structure or attributes of the input data based on the predictions of the capsule in layer *l*, as well as prior knowledge and coupling coefficients.

The overall structure of the Efficient-STCapsNet network is displayed in Figure 2, which contains two main parts. The starting phase (shown in Figure 3) extracts features from the input EEG signals, using convolutional kernels with proper sizes to generate feature vectors containing spatio-temporal attributes. These feature vectors are subsequently mapped into multiple capsules. Upon completion of this process, the basic unit of the network transforms into capsules. Finally, through the self-correlation routing mechanism (shown in Figure 4), each capsule is able to dynamically adjust its attention to other capsules, which enhances the capture of global correlations and thus improves the performance and generalization of the model. Details of the two main parts are described in Section 3.2 and Section 3.3.

### 3.2. Spatio-Temporal Capsule-Generation Block (STCG)

The STCG block is the first part of the proposed Efficient-STCapsNet network, which mainly extracts the spatio-temporal features of the EEG signals through the convolutional layers and maps the features to a high-dimensional space to generate capsules. These generated capsules, called spatio-temporal capsules, are mainly used to represent spatio-temporal feature vectors. Traditional capsule networks eschew pooling operations because they are regarded as leading to information loss in computer vision applications. However, in EEG applications, the continuous nature of brain signals results in significant data redundancy; thus, employing pooling layers to reduce dimensionality can effectively decrease repetitive feature data without losing critical information. Consequently, the proposed STCG block comprises five convolutional layers and two pooling layers. The first layer uses a convolutional kernel of size (1, TI) with F1 filters to extract temporal features, producing F1 temporal feature maps and elevating the data from two dimensions to three dimensions. The second layer employs Depthwise convolution technology, reducing the number of parameters while preserving feature information compared to standard conv2d. This layer uses kernels of size (C, 1), where C = 124, indicating the number of channels. The output is vectors of spatial information merged with temporal features. This layer reduces the data dimensionality from three to two. The third layer is a pooling layer, primarily performing dimension reduction to reduce the data redundancy that exists in EEG signals. The fourth layer employs depthwise separable convolution, combining depthwise and pointwise convolutions. As shown in detail in Figure 3, this layer has two parts. Depthwise convolution uses kernels of size (1, 16) to process signals, while pointwise convolution employs F2 (1, 1) kernels, to amalgamate information across channels. The combination of depthwise and pointwise convolutions enhances the network’s feature extraction capability. The fifth layer performs the pooling operation as well. The final layer uses depthwise convolution to perform feature fusion on the output of the fifth layer, finally transforming these vectors into capsules. Each capsule encapsulates a set of feature vectors with temporal and spatial attributes. On the other hand, in the capsule neural network, the magnitude of the vector is used to represent the probability of the existence of a particular feature. This probability value can be represented more visually by regularizing the magnitude of the vector between 0 and 1. A magnitude close to 1 indicates a high probability of the feature’s existence, while a magnitude close to 0 indicates a low probability. Therefore, it is satisfied by the squash function, as shown in Equation (1).

(1) $$\begin{array}{c} squash\left(s_{n,d}^{l}\right)=\left(1-\frac{1}{e^{\left|\right|s_{n,d}^{l}\left|\right|}}\right)\frac{s_{n,d}^{l}}{\left|\right|s_{n,d}^{l}\left|\right|} \end{array}$$

By means of the squash function, a new matrix, $U_{n^{l},d^{l}}^{l}$, with the same dimensions and properties as $s_{n,d}^{l}$, is obtained, but with a magnitude “squashed” between zero and one.

### 3.3. Self-Correlation Routing Mechanism

The proposed self-correlation routing mechanism is a non-iterative approach that calculates the similarity between capsules. The similarity coefficients among capsules forms a weight matrix according to which the capsules are allocated and aggregated in groups to represent categorized spatio-temporal features. The self-correlation routing mechanism dynamically adjusts the relationship between the capsules to improve the performance of the network, while maintaining its light weight. The overall process of this routing mechanism is shown in Figure 4. The input to the self-attentive routing mechanism is the capsule $U_{\left(n^{l},d^{l+1}\right)}^{l}$ (*n* number of capsules, *d* denotes the dimension of the capsule) created by the STCG block, which contains low-level spatio-temporal features. Firstly, the input capsules are mapped by a learnable transformation matrix, $W_{n^{l},n^{l+1},d^{l},d^{l+1}}$, to higher-level capsules, as shown in Equation (2).

(2) $$\begin{array}{c} {\hat{U}}_{\left(n^{l},d^{l},:\right)}^{l}\;=\;U_{\left(n^{l},d^{l}\right)}^{l}\times W_{\left(n^{l},n^{l+1},d^{l},d^{l+1}\right)} \end{array}$$

In fact, after the transformation, each capsule contains the predicted attributes of the category that it belongs to. Therefore, the final output can be calculated using Equation (3).

(3) $$\begin{array}{c} S_{n,d}^{l+1}\;=\;{\hat{U}}_{\left(:,n^{l+1},:\right)}^{Tl}\times \left(C_{\left(:,n^{l+1}\right)}^{l}+B_{\left(:,n^{l+1}\right)}^{l}\right) \end{array}$$

where $B_{n^{l},n^{l+1}}^{l}$ is a defined trainable parameter, which represents the log priors matrix that contains all the weights obtained through discriminative learning. These priors help create biases that are more in favor of linked capsules. The matrix $C_{n^{l},n^{l+1}}^{l}$ contains all the coupling coefficients generated by the self-correlation mechanism. The two-step process of calculating the coupling coefficient $C_{n^{l},n^{l+1}}^{l}$ begins with the calculation of the self-correlation tensor $A_{n^{l},n^{l},n^{l+1}}^{l}$, using Equation (4).

(4) $$\begin{array}{c} A_{\left(:,:,n^{l+1}\right)}^{l}\;=\;\frac{{\hat{U}}_{\left(:,n^{l+1},:\right)}^{l}\times {\hat{U}}_{\left(:,n^{l+1},:\right)}^{Tl}}{\sqrt{d^{l}}} \end{array}$$

where $A_{\left(:,:,n^{l+1}\right)}^{l}$ is a the symmetric matrix of self-correlation. $d^{l}$ is the dimension of capsules and dividing by $\sqrt{d^{l}}$ accelerates the model converge. Each $A_{\left(:,:,n^{l+1}\right)}^{l}$ contains the scores for each combination of the $n^{l}$ capsules predictions. Therefore, the self-correlation matrix is further computed to obtain the coupling coefficient in Equation (5).

(5) $$\begin{array}{c} C_{\left(:,n^{l+1}\right)}^{l}\;=\;\frac{exp(\Sigma_{n^{l}}A_{\left(:,n^{l},n^{l+1}\right)}^{l})}{\Sigma_{n^{l+1}}exp\left(\Sigma_{n^{l}}A_{\left(:,n^{l},n^{l+1}\right)}^{l}\right)} \end{array}$$

In the Efficient-STCapsNet network, there is a certain coupling coefficient between each capsule in the first layer and each capsule in the second layer, and the sum of these coupling coefficients is 1. To determine the routing weights for each capsule, the initial logarithmic prior probability is added to these coupling coefficients. This process is also the same when there are multiple capsule layers present, which are stacked together to create deeper structures.

### 3.4. Margin Loss Function

Due to the fact that the output of the proposed Efficient-STCapsNet network is a set of vectors, i.e., capsules, margin loss is a loss function specifically designed for training capsule networks. The purpose of using this loss function is to promote the feature representation in network training by minimizing the distance between the predicted capsules and the target capsules. The marginal loss equation is shown in Equation (6).

(6) $$\begin{array}{cc} \zeta_{n} & =T_{n}max(0,m^{+}-\left|\right|u_{n}{\left||\right)}^{2}\\ \; & +\lambda \left(1-T_{n}\right)max(0,||u_{n}\left|\right|-m^{-}{)}^{2} \end{array}$$

where $\zeta_{n}$ represents the margin loss of the n-th capsule, $T_{n}$ represents the target category of the *n*-th capsule, $u_{n}$ represents the output vector of the *n*-th capsule, and $\left|\right|u_{n}\left|\right|$ represents the length of the vector. $m^{+}$ and $m^{-}$ are two hyperparameters that represent the minimum and maximum boundary values between the target and non target categories, respectively. λ is a weight parameter used to balance the losses of two parts.

## 4. Experiment

### 4.1. Dataset

**Figure 5:**
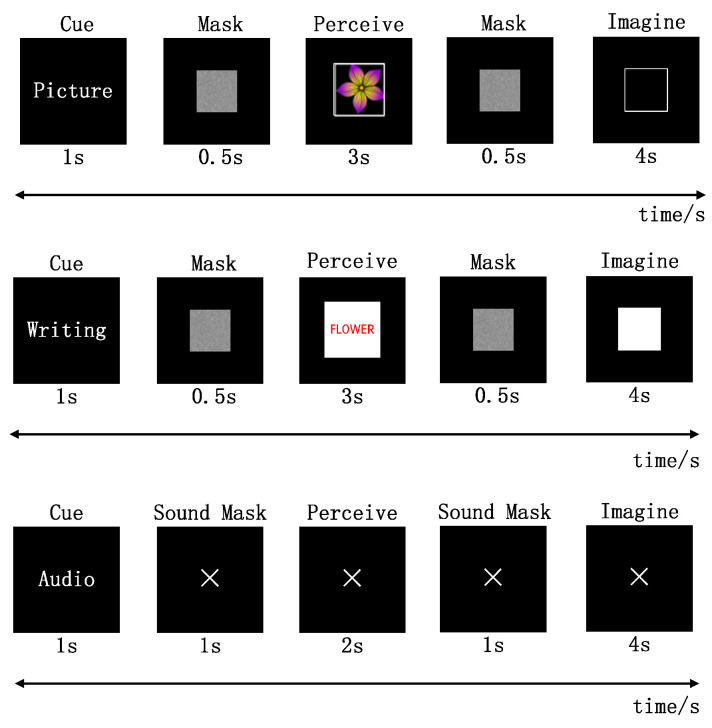
Experimental procedure for generation the dataset of semantic concepts for imagination and perception tasks.

To validate the proposed model, we utilized the publicly available EEG-based BCI Dataset of semantic concepts for imagination and perception tasks from the University of Bath [43]. This dataset used three semantic concepts–-penguin, guitar and flower–-that participants perceived and subsequently imagined in pictorial, orthographic, and audio modalities. Twelve subjects participated in the study, with nine attending one session and three attending two sessions. The EEG brain signals were recorded using a 128-channel ANT Neuro ego Mylab system with a sampling rate of 1024 Hz. We classified the imagination and perception tasks from the EEG signals measured under different sensory conditions for each subject. To ensure fairness in the classification of imagination and perception, all visual condition tasks were standardized to three-second durations, and auditory condition tasks to two-second durations. The specific experimental procedure in the three sensory modalities is shown in Figure 5.

### 4.2. Experiment Settings

In all experiments, we used an NVIDIA GeForce GTX 1080Ti GPU, Python 3.6, and the PyTorch library. The learning rate was set to 0.02, with a batch size of 20, and the model was trained for 120 epochs. The Adam algorithm was selected as the optimizer, and the margin loss was used as the loss function. As this is a binary classification, we consider perception as positive while the imagination is negative. Accuracy was employed as the metric to assess the classification performance of the model, as shown in Equation (7).

(7) $$\begin{array}{c} Accuracy\;=\;\frac{T_{p}\;+\;T_{n}}{T_{p}\;+\;F_{n}\;+\;T_{n}\;+\;F_{p}}\;\times \;100\% \end{array}$$

where $T_{p}$ (true positives) represents the number of samples correctly classified as positive, $T_{n}$ (true negatives) represents the number of samples correctly classified as negative, $F_{p}$ (false positives) is the number of samples incorrectly classified as positive (actually negative), and $F_{n}$ (False Negatives) is the number of samples incorrectly classified as negative (actually positive).

### 4.3. Comparison Experiments and Analysis

To demonstrate the efficacy of the proposed model, we compared it with eight existing algorithms. A brief introduction to these models is provided below:

(1) SVM [43] is a widely used machine learning algorithm. Its fundamental concept is to find an optimal hyperplane in the feature space that separates the features of different categories.

(2) ConvNet [31] is a convolutional neural network designed specifically for EEG classification, which classifies by extracting spatiotemporal features from EEG signals.

(3) CapsNet [37] is to better handle variations in object pose, spatial relationships, and part–whole relationships, thereby addressing the limitations of traditional convolution neural networks.

(4) S3T( Spatial-Temporal Tiny Transformer ) [44] is a spatiotemporal feature learning model for EEG decoding using the Transformer model, proposed by South China University of Technology in 2021.

(5) ConvNeXt [45] is a convolutional neural network architecture developed by Facebook AI Research (FAIR) in 2022, which outperforms the Swin Transformer in image classification tasks.

(6) MTCA-CapsNet [42] is an EEG emotion-recognition method for multi-task learning based on CapsNet and Attention Mechanism.

(7) Conformer [46] is an evolved version of the S3T model that enhances decoding performance through integrating convolutional neural network and Transformer technologies.

(8) LMDA-Net [47] is designed with channel attention and depth attention modules, whose integration significantly enhances classification efficiency in brain-computer interface tasks through effective multidimensional feature integration.

Table 1, Table 2 and Table 3 present the classification accuracy for imagination and perception tasks under the pictorial, orthographic, and audio modalities, respectively. In these tables, the participant number and corresponding session number are connected by an underscore (e.g., 3_3 represents the result of participant 3 in session 3). The highest classification accuracy for each participant is highlighted in bold, while underlines indicate identical experimental results. The proposed model achieved average accuracies of 94.9%, 93.3%, and 78.4% in the pictorial, orthographic, and audio modalities, respectively, outperforming the other compared methods. Compared to other state-of-the-art algorithms, the proposed model achieved improvements in accuracy of 3.6%, 3.5%, and 2.6%, respectively. T-test results show that the proposed algorithm demonstrates significant differences (*p* < 0.05) compared to the advanced algorithms in the pictorial and audio modalities. For the orthographic modality, while there are significant differences (*p* < 0.05) compared to most advanced algorithms, no significant differences were found between the proposed method and MTCA-CapsNet or LMDA-Net. This is mainly due to the fact that in some subjects’ experiments (e.g., subjects 8_3, 15_1, 17_1, 18_1, and 19_1), MTCA-CapsNet and LMDA-Net achieved identical or even higher accuracies than the proposed model. Such overlaps or superior performances resulted in non-significant differences in the statistical analysis. However, the proposed model still showed higher classification performance in the majority of subjects’ experiments, with an average accuracy improvement of 3.5% and 3.8% over MTCA-CapsNet and LMDA-Net, respectively.

To further validate the effectiveness of our method, Table 4 presents the overall average accuracy results across the three sensory modalities for the proposed algorithm compared to advanced algorithms. Even though the differences with other advanced algorithms may not be apparent in individual sensory modalities, the proposed method consistently demonstrates superior overall performance across all modalities, achieving the highest average accuracy and highlighting its comprehensive advantage and robustness in multimodal classification tasks.

**Figure 6:**
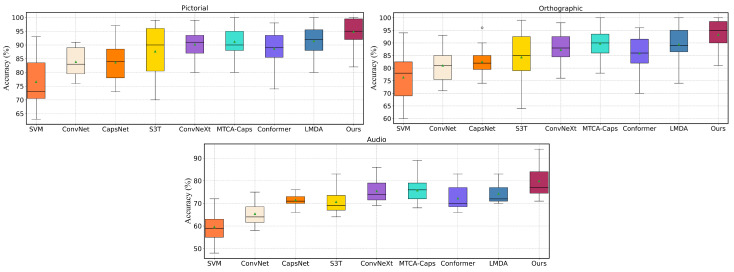
Comparison between the proposed model and state-of-the-art models for overall classification of imagination and perception tasks for all subjects under different sensory modalities.

The box plots in Figure 6 show the distribution of subject classification accuracy for different algorithms across pictorial, orthographic, and audio modalities. Overall, the SVM algorithm performs relatively poorly across all sensory modalities, with accuracy ranging from 70% to 85%. In contrast, the median accuracies of ConvNet, S3T, and ConvNeXt are higher, typically between 80% and 90%. CapsNet’s performance across sensory modalities is slightly inferior to ConvNet and S3T but still maintains around 80% accuracy. MTCA-CapsNet shows excellent performance in the pictorial and orthographic modalities, with a median accuracy close to 90%. Conformer and LMDA also perform well, with median accuracies near or exceeding 85%. Our model stands out in all sensory modalities, achieving the highest and most stable accuracies: over 95% in the pictorial, approximately 95% in the orthographic, and over 75% in the audio. These results indicate that our proposed model exhibits outstanding performance across a variety of tasks. Additionally, the smaller confidence intervals indicate that our algorithm performs very consistently across different subjects, primarily because ST-Capsules include features that distinguish between different EEG signal tasks. Moreover, due to the inherently small dataset size of EEG signals, our model contains fewer overall parameters, which helps reduce overfitting, leading to higher accuracy. The number of parameters in different models is shown in Table 5 and Table 6.

**Figure 7:**
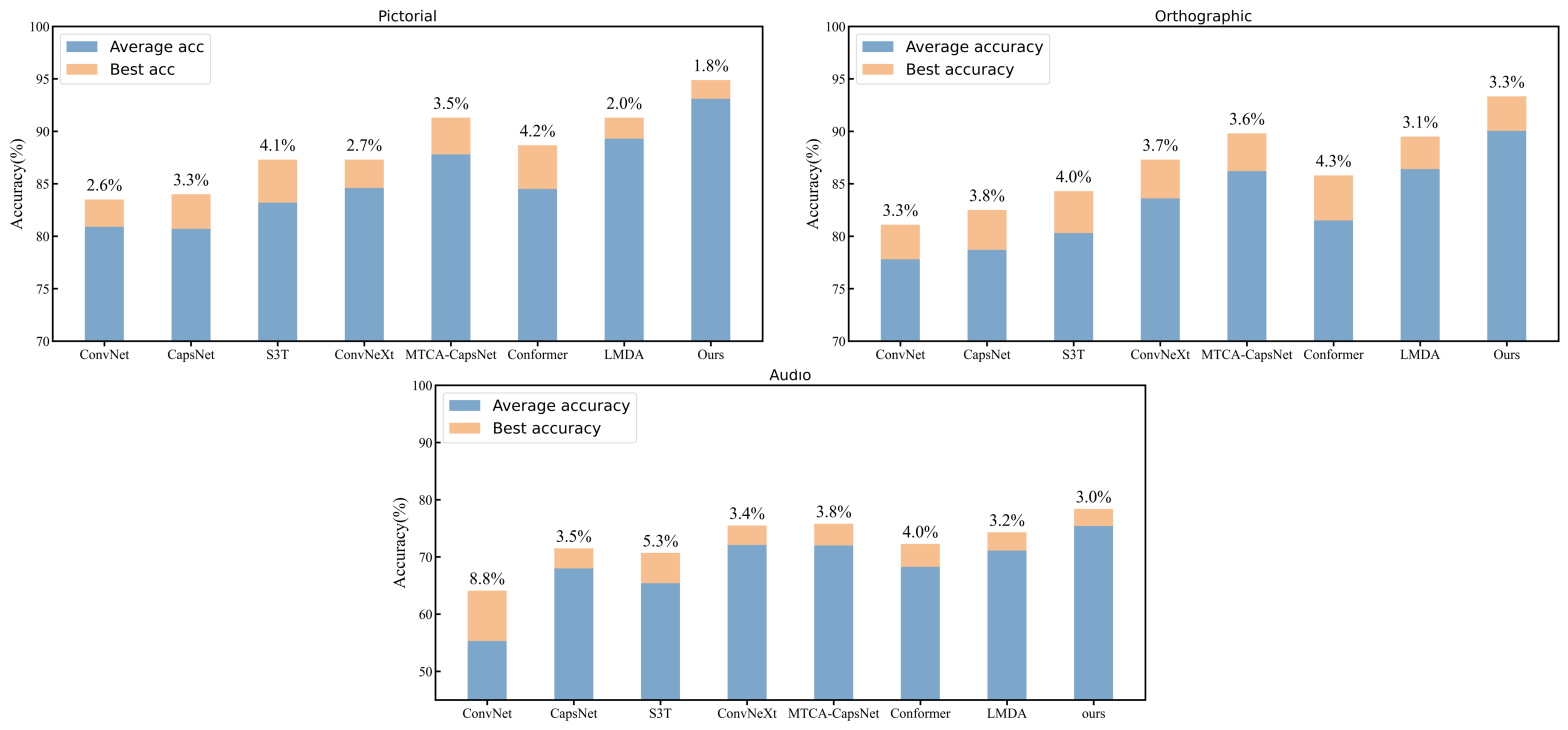
Comparison of the stability of different models.

Additionally, to further verify the model’s stability, we trained and predicted with different models using 10 different random seeds. The data in Figure 7 show that our proposed model outperforms other models in terms of both average and highest accuracy across various datasets, better stability, and smaller accuracy deviation. The proposed method performed particularly well in the pictorial modality. This indicates that our model not only has high accuracy but also strong stability.

### 4.4. Parameter Sensitivity

**Figure 8:**
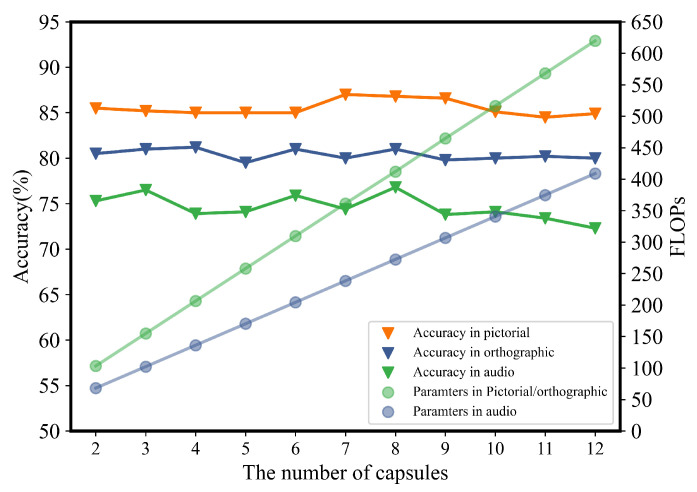
The influence of changes in the number of capsules on accuracy and the amounts of parameters.

**Figure 9:**
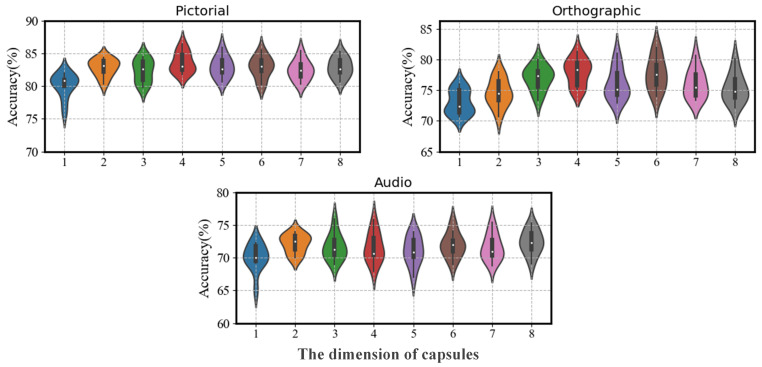
The influence of changes in the dimension of capsules on classification accuracy.

In our proposed model, there are two important parameters: the number of capsules and the dimensions of each capsule. We conducted a detailed analysis to assess the impacts of variations in these two parameters on classification performance. It ought to be noted that there is currently little research specifically on the following aspects: (1) the number of capsules, which determines the number of different computational costs of the model, (2) the dimension of capsules. For the number of capsules, as shown in Figure 8, we explored the impacts of the amount of parameters on the classification accuracy by gradually increasing the number of capsules from 2 to 12. Overall, the change of accuracy for Pictorial and Orthographic tasks under the three different tasks is not significant. Relatively, the differences in audio are the more notable, but the maximum fluctuation is only about 1.2%. These differences are not statistically significant (*p* > 0.05). These results showed that the EEG signal classification was not sensitive to the number of capsules. However, as the parameter curves indicated in Figure 8, an increase in the number of capsules leads to an increase in model complexity (i.e., the amount of parameters), thus reducing the cost-effectiveness of the model. The dimension of the capsules is also a critical factor in capsule networks. Therefore, we compared the impact of capsule dimensions on the model. As shown in Figure 9, we increased the capsule dimensions from 1 to 8 to explore their effect on classification accuracy. As the capsule dimensions increased, classification accuracy improved significantly, but after reaching a dimension of 4, the accuracy tended to saturate and remained relatively stable thereafter.

**Figure 10:**
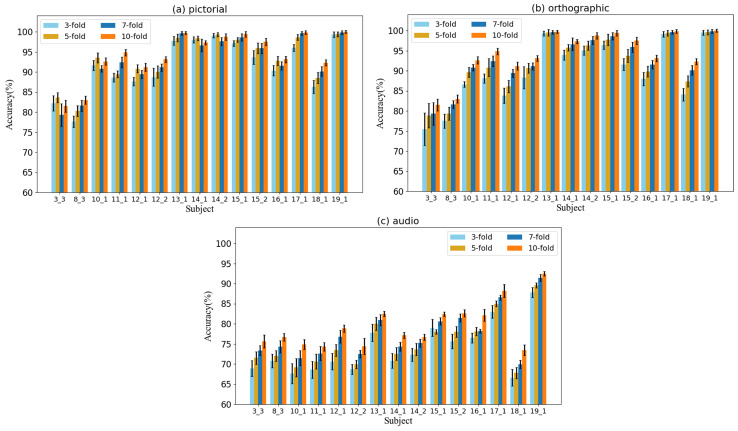
The impact of different cross-validation folds on classification accuracy.

In addition, cross validation can more accurately evaluate the classification performance by avoiding the randomness when relying solely on a single split of the dataset. By dividing the dataset into multiple splits containing training set and validation set, multiple performance indicators of the model can be obtained, which provides a more comprehensive evaluation on the model’s capacity for generalization. Therefore, in order to investigate the impacts of folds in cross validation, we conducted experiments for 3-fold, 5- fold, 7-fold, and 10-fold validations, respectively. The result is shown in Figure 10, where one finds that as the folds increases, the classification performance of the model is improved. Because more folds mean more training validation combinations, this allows the model to learn and evaluate data more comprehensively. However, it should be noted that when increasing the folds, the calculation cost will also increase.

### 4.5. Ablation Study

To further validate the effectiveness of the various improvements in the proposed model, this section presents a detailed discussion. First, we replaced traditional capsules with spatio-temporal capsules. While traditional convolutional layers generate traditional capsules, our improved convolutional layers produce spatio-temporal capsules. We conducted ablation studies across three sensory modalities (pictorial, orthographic, and audio). As shown in Table 7, Table 8 and Table 9, we compared the performance gains achieved by using spatio-temporal capsules instead of traditional capsules under a unified routing method. The results in the first and third rows of these tables indicate that, when using dynamic routing, spatio-temporal capsules significantly outperform traditional capsules, with accuracy improvements of 9.62%, 9.61%, and 6.28%, respectively. Similarly, when employing the self-correlation routing method (as shown in the second and fourth rows), accuracy improvements of 4.59%, 3.63%, and 4.05% were observed. These findings demonstrate that the improved convolutional layer effectively enhances accuracy across all modalities.

**Figure 11:**
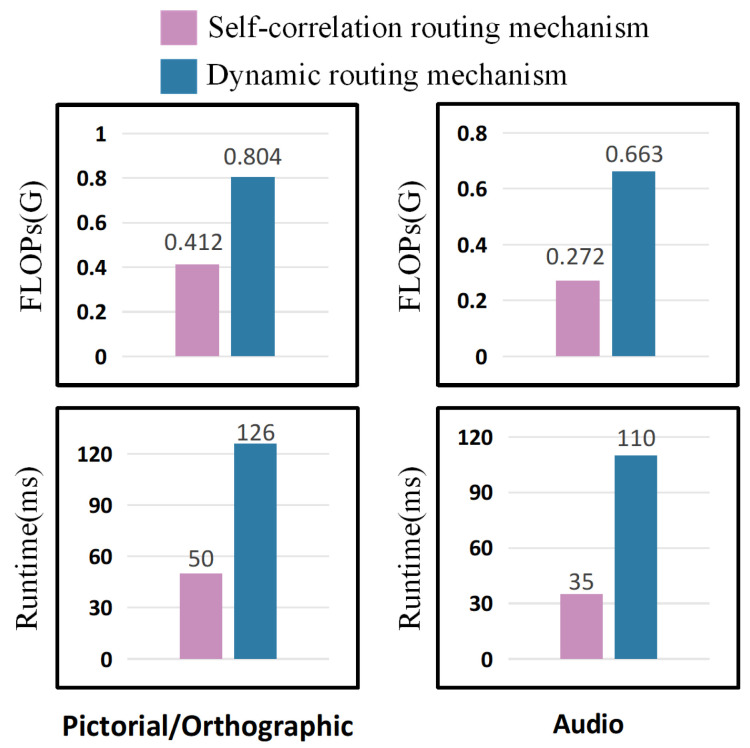
The Comparison of complexity of different routing mechanisms.

Secondly, to address the high computational complexity and lack of inter-capsule connections in the dynamic routing mechanism, we introduced the self-correlation routing mechanism. By comparing the first and second rows, as well as the third and fourth rows of Table 7, Table 8 and Table 9, we observed consistent improvements in average accuracy when using the same capsules. Although the accuracy gains were modest, self-correlation routing offers faster computation and lower complexity, making the model more efficient for classification tasks. As illustrated in Figure 11, the computational complexity of dynamic routing mechanism in terms of FLOPs is nearly double that of self-correlation routing, and it also increases the runtime per epoch. These results clearly demonstrate that our proposed Efficient-STCapsNet model enhances classification accuracy while maintaining computational efficiency.

## 5. Conclusions

To better classify semantic concepts on imagery and perception tasks from EEG signals, this paper proposes a spatio-temporal capsule neural network model with self-correlation routing, which combines a convolutional neural network with a self-correlation routing mechanism. By improving the feature extraction in the convolutional layer and routing mechanisms in the capsule layer, the model captures the spatio-temporal features of EEG signals more effectively and establishes connections between capsules, enhancing classification accuracy and efficiency. Experimental results demonstrate that our method excels in classification tasks across different modalities (pictorial, orthography, and audio), showing significant advantages over existing methods. Additionally, we investigated the impact of capsule quantity and capsule size on model performance. The results indicate that increasing the number of capsules has little effect on classification accuracy; however, increasing capsule size significantly improved the accuracy. Ablation studies show that the model was significantly improved by both using spatio-temporal capsules instead of traditional capsules and adopting the self-correlation routing mechanism. Overall, the proposed model, Efficient-STCapsNet, provided an efficient and reliable solution for decoding semantic concepts in imagination and perception tasks. It significantly improved classification accuracy. We hope the proposed method will contribute to the development of rehabilitation medicine and cognitive neuroscience.

## Figures and Tables

**Table 1:** Performance comparison results of the proposed model and state-of-the-art models in “Pictorial” modalities.

Pictorial	Subjects																
Methods	3_3	8_3	10_1	11_1	12_1	12_2	13_1	14_1	14_2	15_1	15_2	16_1	17_1	18_1	19_1	Avg	p -Vaule
SVM(Baseline)	66%	70%	78%	71%	73%	71%	83%	67%	73%	92%	87%	78%	93%	63%	84%	76.6%	*p* < 0.05
ConvNet	79%	76%	88%	79%	83%	77%	90%	80%	80%	89%	89%	89%	91%	81%	87%	83.9%	*p* < 0.05
CapsNet	75%	73%	85%	83%	84%	78%	88%	78%	75%	97%	87%	89%	93%	80%	90%	83.7%	*p* < 0.05
S3T	70%	78%	83%	75%	78%	76%	95%	99%	98%	90%	90%	87%	94%	83%	99%	86.3%	*p* < 0.05
ConvNeXt	84%	80%	93%	88%	92%	90%	97%	82%	87%	99%	94%	92%	97%	87%	91%	90.2%	*p* < 0.05
MTCA-CapsNet	**88%**	80%	**96%**	85%	86%	89%	92%	88%	95%	100%	92%	90%	95%	86%	100%	90.8%	*p* < 0.05
Conformer	81%	74%	93%	86%	82%	89%	94%	87%	91%	98%	94%	89%	95%	85%	92%	88.7%	*p* < 0.05
LMDA-Net	80%	80%	93%	88%	88%	88%	95%	92%	95%	99%	96%	89%	97%	92%	100%	91.2%	*p* < 0.05
**Ours method**	87%	**82%**	95%	**92%**	92%	**93%**	**100%**	**99%**	**100%**	100%	**97%**	**94%**	**100%**	92%	100%	**94.9%**	-

**Table 2:** Performance comparison results of the proposed model and state-of-the-art models in “Orthographic” modalities.

Orthographic	Subjects																
Methods	3_3	8_3	10_1	11_1	12_1	12_2	13_1	14_1	14_2	15_1	15_2	16_1	17_1	18_1	19_1	Avg	p-Vaule
SVM(Baseline)	60%	66%	80%	76%	78%	73%	86%	66%	72%	81%	82%	84%	94%	64%	83%	76.3%	*p* < 0.05
ConvNet	71%	73%	80%	75%	81%	76%	93%	74%	81%	85%	85%	90%	92%	77%	83%	81.1%	*p* < 0.05
CapsNet	74%	75%	84%	78%	83%	80%	80%	80%	82%	90%	86%	88%	96%	79%	83%	82.5%	*p* < 0.05
S3T	71%	75%	80%	64%	78%	82%	96%	94%	91%	82%	85%	87%	94%	87%	99%	84.3%	*p* < 0.05
ConvNeXt	76%	76%	85%	93%	89%	84%	95%	77%	88%	95%	92%	90%	98%	85%	86%	87.3%	*p* < 0.05
MTCA-CapsNet	78%	**84%**	87%	90%	82%	86%	92%	93%	94%	**100%**	90%	89%	98%	86%	98%	89.8%	*p* > 0.05
Conformer	70%	73%	84%	87%	84%	86%	96%	89%	84%	96%	92%	91%	96%	80%	80%	85.8%	*p* < 0.05
LMDA-Net	74%	76%	89%	89%	84%	84%	97%	91%	90%	97%	93%	89%	100%	89%	100%	89.5%	*p* > 0.05
**Ours method**	**81%**	81%	**91%**	**95%**	89%	**92%**	**100%**	**97%**	**98%**	99%	**95%**	**92%**	100%	89%	100%	**93.3%**	-

**Table 3:** Performance comparison results of the proposed model and state-of-the-art models in “Audio” modalities.

Audio	Subjects																
Methods	3_3	8_3	10_1	11_1	12_1	12_2	13_1	14_1	14_2	15_1	15_2	16_1	17_1	18_1	19_1	Avg	p-Vaule
SVM(Baseline)	48%	59%	59%	57%	62%	53%	63%	59%	55%	55%	63%	63%	72%	55%	71%	59.6%	*p* < 0.05
ConvNet	62%	58%	60%	61%	64%	63%	63%	66%	65%	70%	60%	75%	64%	67%	63%	64.1%	*p* < 0.05
CapsNet	73%	69%	71%	74%	71%	70%	72%	70%	66%	71%	70%	73%	74%	73%	76%	71.5%	*p* < 0.05
S3T	73%	67%	71%	67%	70%	67%	69%	69%	64%	68%	74%	75%	78%	65%	83%	70.7%	*p* < 0.05
ConvNeXt	70%	73%	**74%**	77%	72%	71%	78%	71%	72%	81%	75%	80%	83%	69%	86%	75.4%	*p* < 0.05
MTCA-CapsNet	**77%**	69%	72%	75%	76%	69%	76%	68%	72%	80%	78%	80%	82%	**74%**	89%	75.8%	*p* < 0.05
Conformer	69%	66%	70%	66%	73%	68%	77%	67%	69%	78%	74%	77%	83%	69%	78%	72.3%	*p* < 0.05
LMDA-Net	70%	71%	70%	74%	74%	71%	79%	70%	72%	76%	72%	78%	83%	71%	83%	74.3%	*p* < 0.05
**Ours method**	76%	**74%**	71%	77%	76%	**73%**	**81%**	**76%**	**75%**	**82%**	**81%**	**81%**	**87%**	73%	**94%**	**78.4%**	-

**Table 4:** Comparison of the proposed model with state-of-the-art algorithms shows average results across three sensory modalities (* represents *p* < 0.05).

Modalities	SVM	ConvNet	CapsNet	S3T	ConvNeXt	MTCA- CapsNet	Conformer	LMDA-Net	Our Method
pictorial	76.6%	83.9%	83.7%	86.3%	90.2%	90.8%	88.7%	91.2%	**94.9%**
orthographic	76.3%	81.1%	82.5%	83.7%	87.3%	89.8%	85.8%	89.5%	**93.3%**
audio	59.6%	64.1%	71.5%	70.7%	75.4%	75.8%	72.3%	75.3%	**78.4%**
Average	70.8% *	76.4% *	79.2% *	80.2% *	84.3% *	85.5% *	82.3% *	85.3% *	**88.9%**

**Table 5:** Model complexity comparisons in pictorial and orthographic modalities.

Module	Input Size	FLOP (G)	Total Size (M)
ConvNet	124 × 3073	0.983	120.57
CapsNet	124 × 3073	0.204	278.24
S3T	124 × 3073	0.269	98.80
ConvNeXt	124 × 3073	2.761	120.37
MTCA-CapsNet	124 × 3073	0.207	279.74
Conformer	124 × 3073	1.009	155.83
LMDA	124 × 3073	0.274	156.58
Efficient-STCapsNet (our method)	124 × 3073	0.412	94.74

**Table 6:** Model complexity comparisons in audio modalities.

Module	Input Size	FLOP (G)	Total Size (M)
ConvNet	124 × 2049	0.653	80.33
CapsNet	124 × 2049	0.202	185.79
S3T	124 × 2049	0.177	66.26
ConvNeXt	124 × 2049	1.739	77.61
MTCA-CapsNet	124 × 2049	0.204	186.49
Conformer	124 × 2049	0.671	99.64
LMDA	124 × 2049	0.180	103.52
Efficient-STCapsNet (our method)	124 × 2049	0.272	62.52

**Table 7:** The impact of different module combinations in “Pictorial”.

Traditional Capsules	ST-Capsules	Dynamic Routing	Self-Correlation Routing	Accuracy (%)
✓	×	✓	×	83.66%
✓	×	×	✓	90.32%
×	✓	✓	×	93.28%
×	✓	×	✓	94.91%

**Table 8:** The impact of different module combinations in “Orthographic”.

Traditional Capsules	ST-Capsules	Dynamic Routing	Self-Correlation Routing	Accuracy (%)
✓	×	✓	×	83.66%
✓	×	×	✓	90.32%
×	✓	✓	×	93.28%
×	✓	×	✓	94.91%

**Table 9:** The impact of different module combinations in “Audio”.

Traditional Capsules	ST-Capsules	Dynamic Routing	Self-Correlation Routing	Accuracy (%)
✓	×	✓	×	71.50%
✓	×	×	✓	74.33%
×	✓	✓	×	77.78%
×	✓	×	✓	78.38%

## Data Availability

The EEG datasets used in this paper are available in [43].
